# Measuring health-related quality of life in elementary and secondary school students using the Chinese version of the EQ-5D-Y in rural China

**DOI:** 10.1186/s12889-020-09116-3

**Published:** 2020-06-22

**Authors:** Chen-Wei Pan, Hua Zhong, Jun Li, Chen Suo, Pei Wang

**Affiliations:** 1 School of Public Health, Medical College of Soochow University, Suzhou, China; 2 Department of Ophthalmology, the First Affiliated Hospital of Kunming Medical University, Kunming, China; 3 Department of Ophthalmology, the Second People’s Hospital of Yunnan Province, Kunming, China; 4 School of Public Health, Fudan University, 130 Dong An Road, Shanghai, 200032 China; 5 Key Lab of Health Technology Assessment, National Health Commission of the People’s Republic of China (Fudan University), Shanghai, China

**Keywords:** EQ-5D-Y, China, rural, Health-related quality of life

## Abstract

**Background:**

To measure health-related quality of life (HRQOL) of elementary and secondary school students in rural China using the simplified Chinese version of the EQ-5D-Y.

**Method:**

Both the samples of students were from a school-based cohort study in a county located in southwestern China. The students self-completed the EQ-5D-Y. Feasibility was evaluated according to the percentages of missing values. Known group validity was assessed by comparing the frequency of reporting EQ-5D-Y problems between groups known to differ in health status.

**Results:**

A total of 1728 elementary students and 2116 secondary students were included in the analysis. Their respective mean age was 8.7 (range: 7–15) years and 14.8 (range: 11–18) years, with girls being 45.1 and 50.1%, respectively. The missing values in both samples were quite low. Elementary students were less likely to have problems on‘having pain or discomfort’ and ‘feeling worried, sad or unhappy’ dimensions, but more likely to report problems on the dimensions related to physical functioning. Gender difference in HRQOL was only detected for secondary students in terms of ‘doing usual activities’, ‘having pain or discomfort,’ and ‘feeling worried, sad or unhappy’ (*P* < 0.05 for all). The significant differences in HRQOL were not observed for the other characteristics.

**Conclusions:**

It appears that the EQ-5D-Y is feasible and valid instrument in school-aged children and adolescents in rural China; but it suffers from similar disadvantages to those found in other general populations. The HRQOL distributions measured by the EQ-5D-Y were also provided.

## Background

There have been growing interests in the assessment of health-related quality of life (HRQOL) in children and adolescents in recent years. HRQOL instruments designed for adults may not be suitable for younger respondents as they are in a series of cognitive developmental stages and have different perspectives towards the relative importance of HRQOL dimensions [1, 2]. As a result, a number of instruments considering specific features of children and adolescents have been developed to evaluate generic or disease-specific HRQOL in these age groups [3–9].

The EQ-5D-Y is a generic HRQOL instrument for respondents aged between 8 and 18 years old [8]. It is a child-friendly version of the widely used EQ-5D-3L: its dimensions, wording, and layout were modified from the EQ-5D-3L to ensure relevance and clarity for younger respondents [9]. Compared to other HRQOL instruments for children and adolescents, it has several advantages. First, it keeps the brevity and simplicity of EQ-5D-3L and regarded as easier to complete than other HRQOL instrument, which are important as children’s ability to concentrate is weaker than that of adults. Second, changes in HRQOL from childhood or adolescence into adulthood can be meaningfully traced if the EQ-5D-Y and EQ-5D-3L are used in respective stages of the life course. Third, research has been carried out to convert EQ-5D-Y responses into a preference-based index score for conducting economic evaluations [10]. This would be of great significance as a growing number of studies have been conducted to assess the cost-effectiveness of health interventions for children and adolescents. To date, only two other instruments for children and adolescents can convert HRQOL data into preference scores [5, 11].

The advantages of EQ-5D-Y described above may make it especially suitable in China. For example, its simplicity facilitates its large-scale application (e.g., nationwide or province-wide) as China has a large population of children and adolescents whose characteristics differ in various aspects. Also, its possibility to have preference scores matches the increasing needs of assessing cost-effectiveness of health interventions for children and adolescents in China.

The EQ-5D-Y has demonstrated feasibility, validity, and reliability in both healthy and unhealthy children and adolescents in many countries [8, 12–20]. A few Asian language versions have been available including simplified and traditional Chinese versions; both of them have been validated recently [12, 21, 22]. On the other hand, no study has yet to test it in either rural population or minority population in mainland China, which could have substantial differences in socioeconomic status, lifestyle, and health status in comparison with those living in urban areas or are Han ethnicity. Hence, the study aimed to evaluate its performance in terms of feasibility and validity in school-aged children and adolescents in a rural area of Yunnan province, China. Yunnan is home to various ethnic minority groups. It could be hypothesized that it is also a feasible and valid instrument for measuring their HRQOL. Nevertheless, it may still present ceiling effects and may not differentiate respondents with and without minor health conditions, which were observed in prior studies in general populations [8, 16–20].

## Method

### Study design and population

The data of this analysis were from the follow-up survey of the Mojiang Myopia Progression Study. It is a school-based cohort study aiming to longitudinally observe the onset and progression of myopia as well as other major childhood ocular diseases in school-aged children in rural China. Although the whole cohort study is a longitudinal cohort design, the simplified Chinese version of EQ-5D-Y was only administered in the follow-up survey as it was not finalized during the baseline survey.

The study site (Mojiang) is a county located in Southwestern China with a population of 0.36 million and an area of 5312 km^2^ with a relatively stable demographic structure and similar socioeconomic status to the average of rural China (http://www.stats.gov.cn). The detailed study protocols and some major findings have been described elsewhere [23–26]. Briefly, all the grade 1 students from elementary schools and grade 7 students from secondary schools in Mojiang were invited to participate in the study. The study revealed a lower prevalence of myopia in school students in rural China and found some novel risk factor for myopia. The current analysis focused on the impact of myopia and other factors on generic HRQOL as measured by the EQ-5D-Y among rural Chinese students, which is an extension of our previous work.

In 2016, a total of 2432 elementary students and 2346 secondary students participated in the baseline survey. After 1 year, 2310 (95.0%) elementary students and 2191 (93.4%) secondary students successfully attended the follow-up examination. In the examination, 1728 elementary students (74.8%) and 2114 secondary students (96.5%) attended a self-administered survey including the EQ-5D-Y and demographic questions. The main reason for non-participation was illiteracy given the relatively backward in socioeconomic development of the area. The examination also measured their height, weight, and refractive status.

Ethics committee approval was obtained from the Institutional Review Board of Kunming Medical University. We carried out the study according to the tenets of the Declaration of Helsinki involving human participants and the approved guidelines. Additionally, we obtained written informed consents from at least one parent or legal guardian of each participant.

### EQ-5D-Y

The EQ-5D-Y uses a similar 5-dimenional descriptive system with the EQ-5D but child-friendly wording, referring to mobility (‘walking about’), self-care (‘looking after myself’), usual activities (‘doing usual activities’), pain and discomfort (‘having pain or discomfort’), and anxiety and depression (‘feeling worried, sad or unhappy’). Each dimension only includes one item which has three functioning levels: no problem, some problems, and a lot of problems, resulting in a total of 243 unique health states which can be coded using a 5-digit number. For example, the health state for a person with no problems in ‘walking about’ and ‘looking after himself (herself)’, some problems in ‘doing usual activities’ and ‘having pain or discomfort’, and a lot of problems in ‘feeling worried, sad or unhappy’ can be represented as 11,223.

### Height, weight, and refractive status

Height and weight data were used to calculate BMI. Height was measured to the nearest 0.1 cm by a wall-mounted stadiometer. Participants stood straight, barefoot, with relaxed shoulders and their arms hanging freely. Weight was measured to the nearest 0.1 kg by a scale, with minimal clothing and without shoes. The refractive status of the participant was measured after cyclopedia using an auto refractor (RM-8000; Topcon Corp., Tokyo, Japan). Myopia was defined as spherical equivalent (SE) less than − 0.50 diopter (D).

### Data analysis

Descriptive statistics were reported to describe the characteristics of the two samples. The feasibility reflects whether the EQ-5D-Y is acceptable by the respondents [8]. It was assessed by examining the proportion of missing values on the EQ-5D-Y dimensions. A missing value was defined as a respondent completely leaving out an item. The missing responses were excluded from the respective dimensions, resulting in different numbers of responses for the dimensions in the two samples. The valid responses were kept for further analysis.

The distributions of EQ-5D-Y health profiles were explored for the two samples.

The known group validity was tested by comparing the frequency of reporting problems in the EQ-5D-Y dimensions between the two samples as well as genders and races within each sample. We hypothesized that elementary students would encounter more physical but less psychological problems compared to secondary students [27]; secondary school girls would report more psychological problems than the boys [28]; non-Han ethnicity may suffer from more psychological problems than Han ethnicity [29]. We also examined the difference in frequency between groups known to be different in health status. The groups included: 1) those with and without myopia; 2) those who were underweight, normal weight, and overweight according to BMI level [30]. The EQ-5D-Y may not differentiate the differences between the groups given the nature of the two conditions and study samples [8, 16].Chi-square or Fisher’s exact test were performed whenever appropriate to test whether there were statistically significant difference between the groups in the percentage; and the categories ‘some’ and ‘ a lot of’ were collapsed to a single category (‘any problem’).

All analyses were performed using SAS (version 9.4) at a significance level of 0.05.

## Results

The respective mean age for the elementary school and secondary school sample was 8.7 years and 14.8 years, with girls being 45.1 and 50.1%. The majority of participants in both samples are ethnic minority; while elementary school students had lower BMI levels and less likely to have myopia than secondary school students (Table 1).

**Table 1:** Characteristics of the study subjects

	Elementary School (***n*** = 1728)	Secondary school (***n*** = 2114)
**Gender, % (n)**		
**Boys**	54.9 (948)	49.9 (1054)
**Girls**	45.1 (780)	50.1 (1060)
**Age**		
**Mean (SD)**	8.7 (0.61)	14.8 (0.82)
**Median**	8.7	15.0
**Range**	7–15	11–18
**Ethnicity**		
**Han**	16.6 (287)	16.6 (351)
**Hani**	68.2 (1178)	62.5 (1321)
**Yi**	9.3 (162)	12.0 (253)
**Others**	5.9 (102)	8.9 (189)
**BMI (SD)**	16.2 (2.82)	19.7 (2.89)
**Myopia, n(%)**		
**Presence**	2.5 (43)	30.5 (644)
**Absence**	97.5 (1685)	69.5 (1470)

*SD* standard deviation

The proportion of missing values in both samples was quite low, ranging from 0.75% (‘Look after myself’ and ‘Doing usual activities’) to 0.87% (‘feeling worried, sad or unhappy’) in the elementary students; and 0.28% (‘mobility’) to 1.3% (‘feeling worried, sad or unhappy’) in the secondary students, respectively.

A total of 41 and 72 distinct EQ-5D-Y health states were observed in elementary school and secondary school samples, respectively. The overall ceiling effects (i.e., ceiling effects on all dimensions) were more obvious for the former, with the prevalence of full health state (i.e., 11,111) being approximately 62% in comparison with the prevalence of 41% from the latter sample. Apart from the state, the three most prevalent health states, in order of high to low prevalence, were 11,121 (9.3%), 11,112 (7.4%), and 11,211 (2.4%) in the former sample, and 11,112 (21.6%), 11,122 (17.7%), and 11,121 (7.6%) in the latter sample (Table 2). The worst health state (i.e. 33,333) was only observed in one elementary student.

**Table 2:** Percentage of EQ-5D-Y health profiles by study sample

EQ-5D-Y health state	Elementaryschool, % (N)	Secondary school, % (N)
**11,111**	61.5 (1052)	47.2 (982)
**11,112**	7.4 (126)	21.6 (450)
**11,121**	9.3 (159)	7.6 (159)
**11,122**	5.5 (94)	17.7 (369)
**11,211**	2.4 (41)	–
**11,222**	–	0.82 (17)

The proportions of reported problems in the dimensions varied significantly between the two samples (Table 3). Compared to secondary students, elementary students were less likely to have problems on ‘having pain or discomfort’ and ‘feeling worried, sad or unhappy’ dimensions, but more likely to report problems on the three dimensions related to physical functioning. The largest difference was observed on ‘feeling worried, sad or unhappy’ dimension (i.e. 78.8% vs. 56.1%). High proportion of problems was reported on ‘having pain or discomfort’ and ‘feeling worried, sad or unhappy’ dimensions in both samples. In contrast, problems were reported less frequently on the other three dimensions.

**Table 3:** Percentage of reported problems in the EQ-5D-Y dimensions by study sample

	Elementary School (***n*** = 1728), % (n)	Secondary school,(***n*** = 2114), % (n)
**Mobility (walking about)**		
**No problems**	96.7 (1657)	98.9 (2085)
**Some problems**	2.4 (41)	1.0 (21)
**A lot of problems**	0.93 (16)	0.09 (2)
**Total**	100 (1714)	100 (2108)
***p*-value**^**a**^	< 0.0001	
**Missing values**^**b**^	0.81 (14)	0.28 (6)
**Looking after myself**		
**No problems**	95.6 (1640)	99.1 (2089)
**Some problems**	3.9 (66)	0.76 (16)
**A lot of problems**	0.52 (9)	0.14 (3)
**Total**	100 (1715)	100 (2108)
***p*-value**^**a**^	< 0.0001	
**Missing values**^**b**^	0.75 (13)	0.28 (6)
**Doing usual activities**		
**No problems**	90.3 (1549)	97.2 (2046)
**Some problems**	7.8 (134)	2.4 (51)
**A lot of problems**	1.9 (32)	0.43 (9)
**Total**	100	100
***p*-value**^**a**^	< 0.0001	
**Missing values**^**b**^	0.75 (13)	0.38 (8)
**Having pain or discomfort**		
**No problems**	77.4 (1326)	71.3 (1491)
**Some problems**	20.5 (351)	28.0 (585)
**A lot of problems**	2.2 (37)	0.72 (15)
**Total**	100 (1714)	100 (2091)
***p*-value**^**a**^	< 0.0001	
**Missing values**^**b**^	0.81 (14)	1.1 (23)
**Feeling worried, sad or unhappy**		
**No problems**	78.8 (1349)	56.1 (1171)
**Some problems**	17.6 (301)	42.2 (881)
**A lot of problems**	3.7 (63)	1.6 (34)
**Total**	100 (1713)	100 (2086)
***p*-value**^**a**^	< 0.0001	
**Missing values**^**b**^	0.87 (15)	1.3 (28)

^a^*P*-value was based on Chi-square tests/Fisher-exact tests; ^b^Misssing responses were excluded from the analysis

The differences in reported EQ-5D-Y problems between girls and boys by the samples are shown in Table 4. In elementary school sample, there were no significant divergences on all the dimensions. In secondary school sample, boys experienced significantly more problems on ‘doing usual activities’, ‘but less problems on ‘having pain or discomfort’ and ‘feeling worried, sad or unhappy’ dimensions.

**Table 4:** Percentage of reported problems in the EQ-5D-Y dimensions by gender, by sample

	Elementary School, %(n)			Secondary school %(n)		
	Boys (948)	Girls (780)	***p***-value	Boys (1054)	Girls (1060)	***p***-value
**Mobility (walking about)**			0.196			0.136
**No problems**	96.2 (903)	97.3 (754)		98.6 (1034)	99.2 (1057)	
**Some problems**	3.0 (28)	1.7 (13)		1.3 (14)	0.66 (7)	
**A lot of problems**	0.85 (8)	1.0 (8)		0.1 (1)	0.09 (1)	
**Looking after myself**			0.980			0.476
**No problems**	95.6 (899)	95.6 (741)		99.0 (1038)	99.2 (1051)	
**Some problems**	3.5 (33)	4.3 (33)		0.76 (8)	0.76 (8)	
**A lot of problems**	0.85 (8)	0.13 (1)		0.29 (3)	0	
**Doing usual activities**			0.324			0.008
**No problems**	89.7 (843)	91.1 (706)		96.2 (1008)	98.1 (1038)	
**Some problems**	8.4 (79)	7.1 (55)		3.2 (33)	1.7 (18)	
**A lot of problems**	1.9 (1.8)	1.8 (14)		0.67 (7)	0.19 (2)	
**Having pain or discomfort**			0.766			0.001
**No problems**	77.6 (729)	77.0 (597)		74.5 (774)	68.2 (717)	
**Some problems**	19.7 (185)	21.4 (166)		24.5 (255)	31.4 (330)	
**A lot of problems**	2.7 (25)	1.6 (12)		0.96 (10)	0.24 (5)	
**Feeling worried, sad or unhappy**			0.876			< 0.001
**No problems**	78.9 (740)	78.6 (609)		60.4 (626)	52.0 (545)	
**Some problems**	17.8 (167)	17.3 (134)		38.2 (396)	46.2 (485)	
**A lot of problems**	3.3 (31)	4.1 (32)		1.5 (15)	1.8 (19)	

The differences on all the dimensions between Han and non-Han did not achieve.

statistical significance in both samples (Table 5). The significant differences between respondents with and without a certain condition were not observed for both samples either (Table 6).

**Table 5:** Percentage of reported problems in the EQ-5D-Y dimensions by race, by sample

	Primary School, %(n)			Secondary school, %(n)		
	Han (287)	Non-Han (1441)	***p***-value	Han (***n*** = 351)	Non-Han (1793)	***p***-value
**Mobility (walking about)**			0.157			0.838
**No problems**	97.2 (276)	96.6 (1381)		98.9 (345)	98.9 (1740)	
**Some problems**	2.8 (8)	2.3 (33)		1.2 (4)	0.97 (17)	
**A lot of problems**	0	1.1 (16)		0	0.11 (2)	
**Looking after myself**			0.852			0.848
**No problems**	95.4 (271)	95.7 (1369)		99.1 (346)	99.1 (1743)	
**Some problems**	4.2 (12)	3.8 (54)		0.86 (3)	0.74 (13)	
**A lot of problems**	0.35 (1)	0.56 (8)		0	0.17 (3)	
**Doing usual activities**			0.910			0.394
**No problems**	90.5 (257)	90.3 (1292)		96.9 (338)	97.2 (1708)	
**Some problems**	7.4 (21)	7.9 (113)		2.3 (8)	2.5 (43)	
**A lot of problems**	2.1 (6)	1.8 (26)		0.86 (3)	0.34 (6)	
**Having pain or discomfort**			0.524			0.146
**No problems**	76.3 (216)	77.6 (1110)		75.2 (261)	70.5 (1230)	
**Some problems**	22.3 (63)	20.1 (288)		23.9 (83)	28.8 (502)	
**A lot of problems**	1.4 (4)	2.3 (33)		0.86 (3)	0.69 (12)	
**Feeling worried, sad or unhappy**			0.476			0.235
**No problems**	80.3 (228)	78.5 (1121)		60.3 (208)	55.3 (963)	
**Some problems**	17.4 (49)	17.6 (252)		38.3 (132)	43.2 (749)	
**A lot of problems**	2.5 (7)	3.9 (56)		1.5 (5)	1.7 (29)

**Table 6:** Percentage of reported problems in the EQ-5D-Y dimensions by diagnosis groups, by sample

	Elementary School (***n*** = 1728), %(n)				Secondary school (***n*** = 2144), %(n)				Secondary school (***n*** = 2144), %(n)		
	Under weight (***n*** = 147)	Normal weight *(***n*** = 1435)	Over weight (***n*** = 146)	***p***-value	Under weight (***n*** = 198)	Normal weight* (***n*** = 1713)	Over weight (***n*** = 203)	***p***-value	Myopia, no (644)	Myopia, yes (1470)	***p***-value
**Mobility (walking about)**				0.362				0.569			0.825
**No problems**	94.4 (135)	96.9 (1381)	96.6 (141)		99.5 (197)	98.7 (1686)	100 (202)		98.9 (1449)	98.9 (636)	
**Some problems**	4.2 (6)	2.3 (32)	2.1 (3)		0.51 (1)	1.2 (20)	0		1.0 (15)	0.93 (6)	
**A lot of problems**	1.4 (2)	0.84 (12)	1.4 (2)		0	0.12 (2)	0		0.07 (1)	0.16 (1)	
**Looking after myself**				0.223				0.889			0.543
**No problems**	93.7 (134)	95.5 (1362)	98.6 (144)		99.0 (196)	99.1 (1692)	99.5 (201)		99.1 (1452)	99.1 (637)	
**Some problems**	6.3 (9)	3.9 (55)	1.4 (2)		1.0 (2)	0.76 (13)	0.50 (1)		0.68 (10)	0.93 (6)	
**A lot of problems**	0	0.63 (9)	0		0	0.18 (3)	0		0.2 (3)	0	
**Doing usual activities**				0.830				0.754			0.770
**No problems**	90.9 (130)	90.4 (1289)	89.0 (130)		97.0 (192)	97.3 (1659)	96.5 (195)		97.0 (1420)	97.5 (626)	
**Some problems**	8.4 (12)	7.6 (109)	8.9 (13)		3.0 (6)	2.3 (39)	3.0 (6)		2.5 (37)	2.2 (14)	
**A lot of problems**	0.70 (1)	2.0 (28)	2.1 (3)		0	0.47 (8)	0.50 (1)		0.48 (7)	0.31 (2)	
**Having pain or discomfort**				0.225				0.493			0.465
**No problems**	79.0 (113)	77.3 (1102)	76.0 (111)		76.0 (149)	71.1 (1207)	68.2 (135)		72.0 (1046)	69.8 (445)	
**Some problems**	17.5 (25)	20.8 (297)	19.9 (29)		23.5(46)	28.1 (477)	31.3 (62)		27.4(398)	29.3(187)	
**A lot of problems**	3.5 (5)	1.8 (26)	4.1(6)		0.51 (1)	0.77 (13)	0.51 (1)		0.62 (9)	0.94 (6)	

*Reference group. *P* values indicated overall significance of each comparison. Two separate significance testes were also used to compare the other two groups with the reference group. Bold indicates values that are significantly different from the reference level

## Discussion

In the cross-sectional study of school-aged children and adolescents in rural China, we assessed the performance of the newly developed simplified Chinese version of the EQ-5D-Y. In line with prior studies, the study revealed several similarities of the use of EQ-5D-Y in general populations [8, 16–20]. These included suitable for self-completion indicating by low rate of missing responses, high ceiling effects, and the tendency of reporting more and less problems on dimensions related to psychological well-being [i.e., ‘having pain or discomfort’ and ‘feeling worried, sad or unhappy’] and physical functioning (i.e., ‘walking about’, ‘looking after myself’, and ‘doing usual activities’), respectively.

The percentage of missing responses on the EQ-5D-Y dimensions ranged from 0.28 to 1.3%, which is similar with those in previous studies (generally lower than 1%) [8, 16–20]. We also found that the percentage across the dimensions was stable for elementary students but variant for secondary students. Nevertheless, the quite low missing rates favors the feasibility of the Chinese version of the EQ-5D-Y.

The overall ceiling effects were high in both samples, which is expected. This could be due to two reasons. First, all participants were able to attend school implying healthy enough. Second, the EQ-5D-Y has limited ability to differentiate small and (or) moderate health impairments in general population samples because it only offers three functional levels for response choice [8]. Hence, the development of the EQ-5D-Y with five levels response options (EQ-5D-5LY) is supported [21, 22]. Meanwhile, the ceiling effects was less evident in the secondary school sample compared to the elementary school sample and it also had more health states, indicating a large variability of health status in the sample.

Despite of the high ceiling effects, the EQ-5D-Y was able to discriminate between elementary and secondary students, and between girls and boys in secondary school. Secondary students were more likely to have ‘feeling worried, sad or unhappy problems’ as secondary school is more stressful and students have increased awareness of the stress. While, elementary students tended to encounter more problems on the three physical-related dimensions as they are more vulnerable and incline to have incidents such as bump, scratch, and sprain. Secondary school girls also reported more problems on the two psychological well-being related dimensions than did the boys, which is comparable with those in other studies [28, 31]. Teenager girls generally are more sensitive than teenager boys. The phenomenon could also be explained by an integrated model consisting of several factors [28].

The differences in HRQOL were not detected in terms of race and between respondents with and without a condition. The possible differences between Han and non-Han ethnicity may be masked in the analysis as the majority of participants are ethnic minority. Although the symptoms incurred from myopia or abnormal weight might be minor compared to those from severe conditions such as acute lymphoblastic leukemia, this yet implies the EQ-5D-Y may not be sensitive to them, which is similar to the findings reported in previous studies [8, 16]. Indeed, studies using the PedsQL 4.0 in China found that the two conditions did have significant lower HRQOL scores than healthy children and adolescents in one or several aspects [24, 30]. Hence, it has to be used with caution in children and adolescents with minor or no health conditions.

The study has several limitations. First, the study participants were recruited in the general population locally from Mojiang County. The HRQOL distribution may not represent other rural regions in China such as areas with less proportion of non-Han ethnicity. Second, the feasibility was tested only by counting the missing rate. Thus, the time taken to finish the EQ-5D-Y cannot be recorded and compared to the recommended time. Third, no other valid HRQOL instruments were used in our study, making it impossible to comprehensively test the divergence/convergence validity of EQ-5D-Y. Hence, future studies are warranted to systematically evaluate the property in Chinese children and adolescents. Fourth, the reliability (e.g., internal consistency reliability and test-retest reliability) and responsiveness were not assessed due to the cross-sectional design.

## Conclusions

In conclusion, it appears that the EQ-5D-Y is a feasible and valid instrument for measuring HRQOL in school-aged children and adolescents in rural China; but it also suffers from similar disadvantages to those detected in other general populations. The HRQOL information of the participants may also be useful in future studies comparing HRQOL and its factors among different regions in China.

## Data Availability

Please contact author for data requests.

## Abbreviations

BMI: Body mass index
HRQOL: Health-related quality of life
SE: Spherical equivalent
